# Honeybee Colony Thermoregulation – Regulatory Mechanisms and Contribution of Individuals in Dependence on Age, Location and Thermal Stress

**DOI:** 10.1371/journal.pone.0008967

**Published:** 2010-01-29

**Authors:** Anton Stabentheiner, Helmut Kovac, Robert Brodschneider

**Affiliations:** Institut für Zoologie, Karl-Franzens-Universität Graz, Graz, Austria; University of Arizona, United States of America

## Abstract

Honeybee larvae and pupae are extremely stenothermic, i.e. they strongly depend on accurate regulation of brood nest temperature for proper development (33–36°C). Here we study the mechanisms of social thermoregulation of honeybee colonies under changing environmental temperatures concerning the contribution of individuals to colony temperature homeostasis. Beside migration activity within the nest, the main active process is “endothermy on demand” of adults. An increase of cold stress (cooling of the colony) increases the intensity of heat production with thoracic flight muscles and the number of endothermic individuals, especially in the brood nest. As endothermy means hard work for bees, this eases much burden of nestmates which can stay ectothermic. Concerning the active reaction to cold stress by endothermy, age polyethism is reduced to only two physiologically predetermined task divisions, 0 to ∼2 days and older. Endothermic heat production is the job of bees older than about two days. They are all similarly engaged in active heat production both in intensity and frequency. Their active heat production has an important reinforcement effect on passive heat production of the many ectothermic bees and of the brood. Ectothermy is most frequent in young bees (<∼2 days) both outside and inside of brood nest cells. We suggest young bees visit warm brood nest cells not only to clean them but also to speed up flight muscle development for proper endothermy and foraging later in their life. Young bees inside brood nest cells mostly receive heat from the surrounding cell wall during cold stress, whereas older bees predominantly transfer heat from the thorax to the cell wall. Endothermic bees regulate brood comb temperature more accurately than local air temperature. They apply the heat as close to the brood as possible: workers heating cells from within have a higher probability of endothermy than those on the comb surface. The findings show that thermal homeostasis of honeybee colonies is achieved by a combination of active and passive processes. The differential individual endothermic and behavioral reactions sum up to an integrated action of the honeybee colony as a superorganism.

## Introduction

Among social insects, the cavity nesting honeybee species *Apis mellifera* and *A. cerana* display the most advanced regulation of the nest climate [1]. Of these two species, *Apis mellifera* is the more intensely investigated concerning thermal homeostasis of the colony. The endothermic heat production and the insulation of the breeding cavity allows the bees to regulate the brood nest temperature within the range of 32–36°C [e.g.], [ 2]–[10], or to survive cold winters [11], [12]. As the honeybee brood is extremely stenothermic, accurate temperature regulation is indispensable for its proper development [4], [13]. While eggs and larvae (in open brood cells) can tolerate lower temperatures for some time, the pupae (in sealed brood cells) are very sensitive to cooling. If they remain too long below 32°C there is a high incidence of shrivelled wings and legs, and malformations of the abdomen [4], and adults may suffer from neural and behavioral insufficiencies [14]–[17]. Accordingly, the accuracy of thermoregulation is high in the presence of brood [3], [4], [9], [10], and much more variable and generally lower in broodless colonies [8], [18].

The brood itself has a low metabolic rate at young ages [19]–[21]. As it lacks regulatory ability and does not provide enough heat on its own, it would not be able to achieve thermal constancy in a variable environment. Warming of the brood has therefore to be accomplished by the worker bees. This warming behavior is released by chemical stimuli and physical properties of the brood. Sealed cells are more attractive than open ones [13], [20]. If the hive is in danger of being overheated the bees cool it by fanning, and collected water is spread on the combs [20], [22]–[24].

Much research on the thermoregulation of breeding honeybee colonies concentrated on the colony as a whole “superorganism” [25], [26], describing aspects of the hive microclimate like the temperature of the brood nest and the combs, the air temperature and/or humidity between them, the temperature gradient across the colony at various ambient conditions, and diurnal rhythms of hive temperature and metabolic rate [e.g. 3]–[5], [8], [18], [20], [25], [27]. Investigations of this type, however, neglect the fact that thermal homeostasis of a honeybee colony is the result of the cooperation of thousands of individuals in a multi-factor regulatory system. On the individual level there is profound knowledge on the respiration and thus heat production of the eggs [28], the larvae [19], [21], and of resting and active bees of different ages [29]–[31]. Principles of the individual brood warming behavior by active heat production (endothermy) *via* the thoracic flight muscles were investigated in bees heating brood cells from outside [9], [32]–[34], and in bees heating the brood from within cells [10]. However, any comprehensive approach of the mechanisms of colony thermoregulation has to include the contribution of ectothermic bees to colonial heat production. In order to understand the relative importance of the regulatory mechanisms, parallel investigation of as many variables as possible is necessary. We here provide quantitative experimental data of honeybee body temperature in reaction to environmental temperature changes. This allows detailed analysis of the regulatory mechanisms of colony temperature homeostasis driving dynamics and relative frequency of endothermy (with active heat production) and ectothermy (no heat production beyond the resting level) of individuals in different hive locations. The use of infrared thermography made possible thermal investigations on a high number of bees without behavioral impairment.

Honeybee workers were reported to heat brood combs also from within cells [10], [35]. However, the importance of this behavior in relation to endothermic activity of bees on combs is unknown. We here not only analyze the frequency of cell visitation in dependence on changes of environmental temperature but also present a comparison of the frequency of endothermy between bees in cells and on combs. Special emphasis is given to the analysis of the direction of heat flow in cells visited by bees.

Temporal polyethism (age-related division of labor) within honeybee colonies usually addresses the visually observable activities like cell cleaning, nursing, food processing, guarding and foraging [see e.g. 36]–[43]. Harrison [32] was the first to investigate the contribution of individual workers of different age to another important task, colonial heat production. Applying a high cold stress to single combs, he was not able to find age-dependent differences in the intensity of endothermy of hive bees older than 2 days. Only 1-day-old bees displayed less intense endothermy. However, the age distribution of endothermic activity might well be different at less extreme temperatures. We here report extended measurements on this topic, including very young bees (age <24 h), with emphasis of the relative abundance of both endothermic and ectothermic bees in various age classes from emergence to the foraging age.

## Materials and Methods

### Colonies, Bee Treatment and Experimental Procedure

Experiments were carried out in observation hives with two vertically aligned combs which were covered by plastic films transparent to infrared radiation. The bees could leave and enter the hive at their will through an about 1.5 m long transparent plastic tube with 5 cm inner diameter. The cold stress of the test colonies was varied by placing them in an air stream at experimental temperatures (T_exp_) of 15, 20, 25, 30 or 34°C. Air temperature near the bees (T_a_) was measured by a mesh of 20 thermocouples on each side of the colony, mounted at a height of 5–9 mm above the combs. Actual bee position on the combs was determined relative to a wire mesh with 3.1×3.4 cm rectangles mounted at a height of 10 mm above the combs. By dividing each mesh rectangle into 5 subsections (a-d and center) the bee position could be determined with a resolution of about ±10 mm. Together with laboratory temperature and humidity, and experimental air stream and outside temperature, thermocouple data were stored every 10 s on a laptop computer via a network of one ALMEMO 5590-2 (40-channels) and two ALMEMO 2290-8 data loggers (5-channels each; Ahlborn).

Observation colonies were stocked with about 4000–5000 workers and a queen from a standard colony (*Apis mellifera carnica*). Sealed brood combs from several standard colonies were transferred to an incubator and kept there at 34°C. Every second morning 150 bees that had emerged during the night before were marked with a color code for age identification by small paint dots (‘Edding 751 paint marker’) on the margin of abdomen and/or thorax to avoid disturbance of thermographic body temperature measurements, and added gently to the upper comb. After 4 weeks of adding bees, where the colonies had time to settle in and establish a brood nest, the first measurements were started. Freshly added bees were allowed to equilibrate and distribute for at least two hours before measurement. Data are from three colonies and 36 measurement sessions (years 2000–2002).

All marked bees of any age found on one side of the colony were measured by scanning the combs with an infrared (IR) thermographic camera (see below). The thermographed colony side was changed regularly between measurement days. As at low experimental temperatures (15 and 20°C) bees hang in several layers on the combs, we first measured all marked bees in the outermost layer. During the second scan the outer bees were gently pushed aside to get a view to the bees and the comb below. From these measurements the age of the youngest bee group was only known to be in the range of 3–27 h. To get a finer age resolution of very young bees, a second series of experiments was conducted (2 replicates at T_exp_ = 15°C experimental temperature, and one at 20, 25, 30 and 34°C each). For each of these experiments, we marked 100 bees 0–1 h after emergence in the morning, and added them gently to the center of the upper comb. Starting after one hour of equilibration, the body surface temperatures just of the bees of this second series were measured at 10:00, 14:00 and 20:00 hours, on both sides of the colony. This way, at each T_exp_ the youngest bees were always of an age of 1–3 h during measurement. Older bees which had been introduced on earlier days (at different T_exp_) were included. As especially the young bees were often seen to slip into open cells, a separate scan was made where all bees in cells were touched on the abdomen with a blunt needle until they left the cell. Their body temperatures, together with the temperatures of rim and base of the cells where they had been in, were measured immediately after their emergence (within 0–2 s after leaving the cell). In addition to marked bees, all naturally emerged unmarked bees coming out of cells were measured (unmarked bees and ‘nn’ in Figures).

### Body and Comb Temperature Measurement

**Figure 1:**
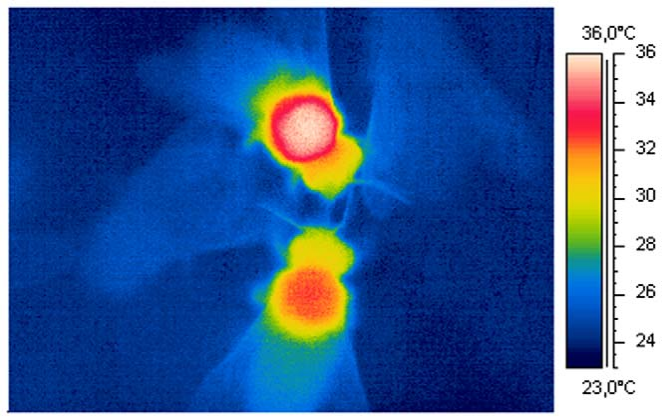
Close-up thermogram of honeybees. Left and right: ectothermic bees (light blue: T_thorax_ = 25.2°C and 24.9°C). Top and bottom: endothermic bees (white: T_thorax_ = 35.6°C; orange: T_thorax_ = 32.4°C). T_a_ = 25°C, T_cell rim_ ∼24°C. Measurement performed in a peripheral area of an observation hive during high cold stress (T_exp_ = 20°C)**.**

Dorsal honeybee body and comb surface temperature was measured thermographically with a ThermaCam SC2000 NTS equipped with a close-up lens (Fig. 1; FLIR, Inc.; 320×240 pixel sensor, thermal resolution <0.1°C). Thermography allowed measurement of body surface temperature without behavioral impairment. The infrared (IR) camera was calibrated for offset errors against an AGA1010 reference radiator visible in the IR picture via a highly reflective aluminium mirror, or against a custom-made peltier-driven miniature reference source of known temperature and emissivity. Non-uniformity within thermographic pictures was corrected by a VBA macro which took into account differential within-picture drift in dependence on the bees' position in the picture, between the camera's internal shutter calibrations. Attenuation of the IR radiation by the plastic films covering the colonies was compensated for by changing the atmospheric transmission value accordingly during evaluation. Using an infrared emissivity of 0.97 of the insect cuticle and of 0.95 of the comb wax, surface temperature was measured to the nearest 0.8°C by this procedure [44].

Thermographic data were stored digitally with 14 bit resolution on a DOLCH FlexPac-400-XG portable computer at a rate of 2 frames s^−1^ for the bees on the combs, and at 10 frames s^−1^ for the second experimental series with very young bees and the bees from within the cells. In addition, thermographic scenes were stored in real time (25 frames s^−1^) on a SONY Hi8 Video Walkman, together with the spoken commentary on bee marking (age code), position in the wire mesh raster, behavior, and the prevailing type of comb cells at the bee position (open and closed honey or brood cells, pollen cells and empty cells). At the same time we pointed at the bee of interest with a needle which was visible in the video. After the measurements, once per day, the exact ranges of brood cells, honey and pollen stores (open and closed where applicable) and empty cells were drawn on a transparent plastic film laid over the side(s) of the hive where the bee temperatures had been measured.

### Data Evaluation

The bees' behavior was judged from the thermographic video sequences and the spoken commentary on the tapes. Evaluation of the surface temperatures of head (T_head_), thorax (T_thorax_) and abdomen (T_abdomen_), and of cell rim and cell base (T_cell rim_, T_cell base_) was done from the files after the measurements, with AGEMA Research software (FLIR) controlled by a custom programmed Excel VBA macro. Temperature data necessary for exact temperature calculation at the thermographic measurement points were automatically extracted from the logger files and interpolated temporally by this macro. Local air temperature at the actual positions of the bees (T_a_) was calculated by triangular interpolation between adjacent thermocouples with a different Excel macro, which extracted the necessary temperature data from the logger files.

Statistics was done with the Statgraphics package (Statistical Graphics Corporation) or with self written Excel sheets (Microsoft Corporation) according to Sachs [45]. Correlations were calculated with Statgraphics or ORIGIN (OriginLab Corporation). For Chi^2^ statistics the significance level was adjusted according to the Bonferroni correction for multiple comparisons wherever applicable [45].

## Results

### Location

**Figure 2:**
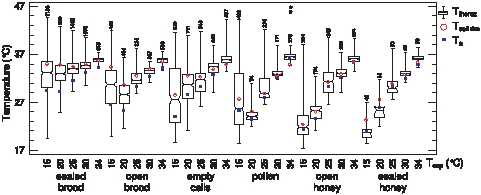
Worker honeybee thorax temperature on different locations in the observation colonies in dependence on cold stress (T_exp_). Box plots: median T_thorax_ with 1^st^ and 3^rd^ quartile, maximum and minimum. Notches which do not overlap indicate significant differences (P<0.05). T_cell rim_ and T_a_: medians. Numbers  =  n (T_thorax_). * are two bees during intense examination by hivemates [45], [46].

Fig. 1 shows a typical thermogram of ectothermic and endothermic bees. The dorsal thorax surface temperatures (T_thorax_) of marked bees on different locations in the observation hives is shown in Fig. 2. The body surface temperature of the bees varied in a wide range. Both the lowest and the highest T_thorax_ were measured at an experimental temperature (T_exp_) of 15°C: minimum T_thorax_ = 17.5°C (T_head_ = 17.1°C, T_abdomen_ = 17.5°C, T_a_ = 19.8°C, T_cell rim_ = 18.9°C) and maximum T_thorax_ = 44.5°C (T_head_ = 41.4°C, T_abdomen_ = 39.5°C, T_a_ = 31.0°C, T_cell rim_ = 35.9°C). Two exceptionally hot bees on pollen stores at T_exp_ = 34°C with a T_thorax_ of 46.9 and 47.3°C (asterisks in Fig. 2) were excluded from further analysis because they originated from bees heating their thorax up during intense examination by hivemates [46], [47]. In general, an increasing cold stress (i.e., a decreasing T_exp_) affected the bees' body surface temperature the least on sealed brood, and somewhat more on open brood. An increasingly higher dependence of T_thorax_ on T_exp_ was observed in bees on empty and pollen cells, and even more on open and sealed honey cells (Fig. 2).

At T_exp_ = 34°C the median T_thorax_ was slightly higher than T_cell rim_ and clearly higher than the local ambient air temperature (T_a_) on all locations (Fig. 2). At lower T_exp_, T_thorax_ was lower than T_cell rim_ and higher than T_a_ both on sealed and open brood. This was only partly the case on empty and pollen cells. At T_exp_ <25°C T_thorax_ was mostly higher on sealed and open brood in comparison with non-brood areas. Bees on open and sealed honey cells were similar in their close decrease of T_thorax_ with T_cell rim_ and T_a_. They differed from each other in so far as at T_exp_ below 30°C the bees' median T_thorax_ was always above T_a_ and partly above T_cell rim_ on open honey, but always below T_a_ and T_cell rim_ on sealed honey (Fig. 2). The variability of body temperature increased considerably with cold stress. This means that at low temperatures an increasing amount of bees actively increased the body temperature noticeably above T_a_. Active heat production was observed the least on open and sealed honey cells. A detailed analysis of data showed that ANOVA was only partly permissible due to not normally distributed data, considerable differences in variance and type of distributions to be compared, or missing linear relationships of parameters. A three factor ANOVA with brood status as the main effect and T_exp_ and worker age as covariates showed that, if each factor was measured having removed the effects of all other factors, T_exp_ had by far the highest influence on T_thorax_-T_abdomen_ of bees on combs (in intercomb spaces) (mean square F-ratios_1.76816_: 1103.83 for T_exp_, 164.49 for age, and 14.99 for brood; P<0.0001). Median Worker T_thorax_-T_abdomen_ was slightly but significantly higher on brood (0.3°C) than on non-brood areas (0.2°C) (P<<0.0001, Kruskal-Wallis test).

### Age and Abundance

**Figure 3:**
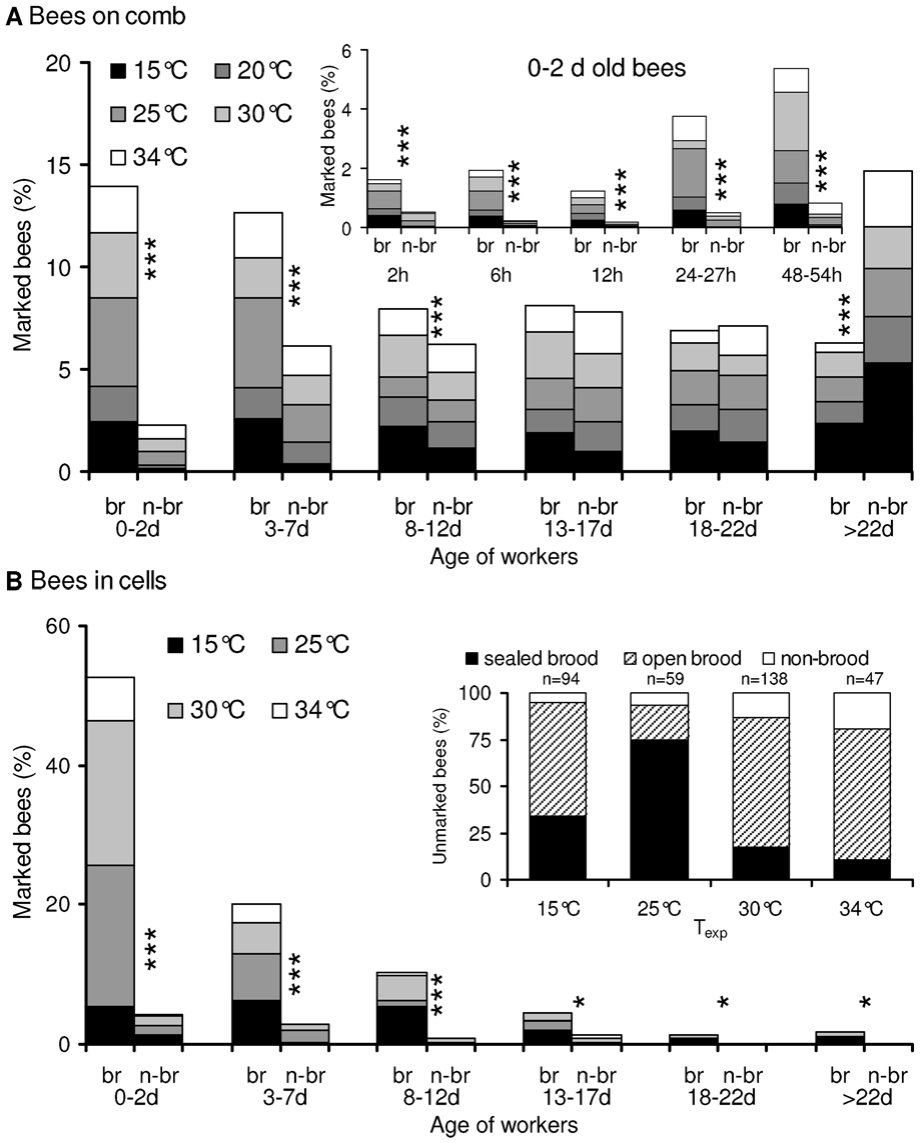
Frequency of honeybees of different ages on brood (br) and non-brood (n-br) areas. Shading of bars  =  thermal stress (T_exp_). (A) Marked bees on combs; 100% = 12732 bees (measurements) of all age classes. Insert: detailed analysis of 0–2 d old bees. (B) Bees in cells; 100% = 350 bees of all age classes. Percentage of bees in brood nest cells per age class (young to old): 92.5 (184 of 199), 87.5 (70 of 80), 92.3 (36 of 39), 76.2 (16 of 21), 100.0 (5 of 5), 100.0 (6 of 6); no significant differences between age classes (P>0.05, Chi^2^ = 0.00–6.08). Insert: distribution of unmarked bees on brood and non-brood areas (‘nn’ in Figs 7, 8); n = bees = 100% (per T_exp_). (A, B) Significant differences between brood and non-brood: * P<0.05, *** P<0.001, Chi^2^ test.

Marked bees were present both on the combs and inside cells. In general, considerably more bees were on the combs than inside cells. Concerning the bees on the combs the relative distribution of bees between brood and non-brood areas differed in dependence on age (Fig. 3A, Fig. S1). The young bees (0–2 d) clearly preferred the brood nest. With increasing age this preference disappeared, with an even distribution in the 13–17 d old and 18–22 d old bees. In bees of forager age (>22 d) the relation reversed, bees were more than twice as abundant outside the brood nest as on it. This trend was similarly visible at low and high cold stress (at all T_exp_; Figs 3A, S1).

Most of the bees inside cells were seen in the brood nest area although many open cells were not occupied.. This was similar in the naturally emerged (unmarked) and the added (marked) bees, even at high temperature (T_exp_ = 34°C; Fig. 3B). With increasing cold stress more bees entered empty cells in the brood nest area. Especially in young bees (<2d) this preference was very pronounced. The tendency to visit cells was much lower in older bees both on the brood nest and outside it (Fig. 3B). Unlike in the bees on combs (in intercomb spaces; Fig. 3A), however, the relation of bees in cells of brood and non-brood areas was similar in all age classes up to the forager age (see legend of Fig. 3B).

### Age and Body Temperature

**Figure 4:**
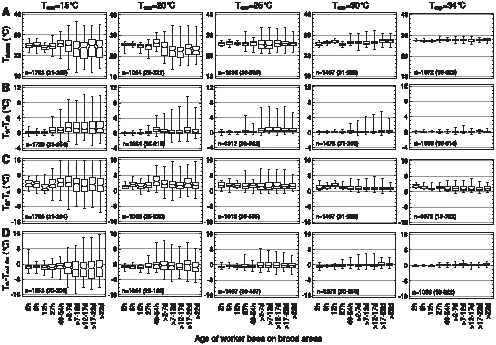
Body temperature and temperature excess of workers of different ages on brood in dependence on thermal stress (T_exp_). Box plots: median with 1^st^ and 3^rd^ quartile, maximum and minimum of (A) T_thorax_, (B) T_thorax_-T_abdomen_, (C) T_thorax_-T_a_, and (D) T_thorax_-T_cell rim_. n = measurements (bees) (range in parenthesis).

Fig. 4 shows the age dependence of the thorax surface temperature (T_thorax_) and its relation to the temperature of the abdomen (T_abdomen_), local ambient air (T_a_) and comb (T_cell rim_) on the brood nest. The medians of T_thorax_ of the investigated age classes were 35.2–36.0°C when T_exp_ was 34°C (Fig. 4A). Median T_thorax_ decreased not below 31.4–33.6°C when a very high cold stress acted on the hive at T_exp_ = 15°C. At all T_exp_ conditions the bees younger than 12 h showed the lowest variation of T_thorax_ (Fig. 4A). In older bees the variation of T_thorax_ increased considerably, especially during high cold stress (low T_exp_), reaching the maximum at about 7 days. This was caused by a higher degree of endothermy (see below) and a higher variation of T_a_ in the locations where the older bees were seen. In the very young bees (<12 h) the median T_thorax_ decreased less in response to an increase of cold stress than in the older age classes, because they preferably stayed in the well regulated brood nest (Fig. 3).

ANOVA indicated a dependence of the temperature excess T_thorax_-T_abdomen_ on age both on brood and non-brood areas if the effects of T_a_ and T_cell rim_ were removed (mean square F-ratios_1,47078_: 6.57 for brood and 5.33 for non-brood; P<0.0001). On the brood nest, median T_thorax_-T_abdomen_ was quite similar and at a low level in all age classes at T_exp_ = 34°C (0.10–0.18°C, Fig. 4B). An increase of cold stress influenced T_thorax_-T_abdomen_ not much in the very young bees (≤12 h) but considerably more in the older ones (maximum effect reached in the 2–7 d old bees; P<0.0001 at T_exp_ <30°C, Mann-Whitney U test). Especially in the age classes >12 h at least 75% of the bees had a thorax which was warmer than the abdomen (see 1^st^ quartiles in Fig. 4B). The T_thorax_ was in most bees higher than the T_a_ at T_exp_ = 34°C, with no visible tendency to any age dependency (Fig. 4C). During high cold stress (T_exp_ = 15°C) the median T_thorax_-T_a_ increased in general, with mostly more than 75% of an age class showing a T_thorax_ higher than T_a_ (see 1^st^ quartiles in Fig. 4C). The variation of T_thorax_-T_a_ increased with cold stress in bees older than 12 h. The T_thorax_ was quite similar to the comb surface (T_cell rim_) at T_exp_ = 34°C (Fig. 4D). Variation of data was again slightly higher in bees older than 12 h. With increasing cold stress the thorax became increasingly cooler than T_cell rim_, with a median difference of up to −2.8°C in the >7–12 days old bees at T_exp_ = 15°C. In parallel, in the bees older than 12 h also variation increased. Nevertheless in these age classes a considerable amount of bees (in most cases more than 25%, (see 3^rd^ quartiles in Fig. 4D) remained warmer than the comb surface even during very high cold stress.

Outside the brood nest (Fig. S2) the T_thorax_ was similar to that on the brood nest in the general trend (especially at high T_exp_) but different in detail. As bees younger than 54 h were not very abundant outside the brood nest (Fig. 3A) a comparison with the brood nest and between age classes was not possible at all T_exp_ because of low sample sizes (despite a total of 12732 measurements). In general, T_exp_ had a stronger effect on body temperature outside than on the brood nest (Fig. 2). At T_exp_ = 15°C medians were 22.1–33.6°C in comparison to 31.4–33.6°C on the brood nest. Especially in the bees older than 54 h T_thorax_ decreased stronger with increasing cold stress than on the brood nest (median T_thorax_ at T_exp_ = 15°C: 22.1–25.7°C outside the brood nest, and 31.4–33.0°C on the brood nest). T_thorax_-T_abdomen_ of bees outside the brood nest, on food and empty cells, was similar to that of bees on the brood nest for all ages and all T_exp_ conditions (medians at T_exp_ = 15°C: 0.32–1.95 versus 0.12–1.32°C, respectively). T_thorax_-T_a_ was on average somewhat smaller outside the brood nest than on it (medians at T_exp_ = 15°C: −0.02–5.9 versus 1.44–4.34°C). T_thorax_-T_cell rim_ was higher outside the brood nest than on it at high T_exp_ (34 and 30°C), especially in bees older than 54 h. At a low T_exp_ of 15°C T_thorax_ was less frequently below T_cell rim_ outside the brood nest than on it (medians at T_exp_ = 15°C: −4.07–+0.47 outside versus −2.82–−0.94°C on the brood nest).

### Endo- and Ectothermy

**Figure 5 pone-0008967-g005:**
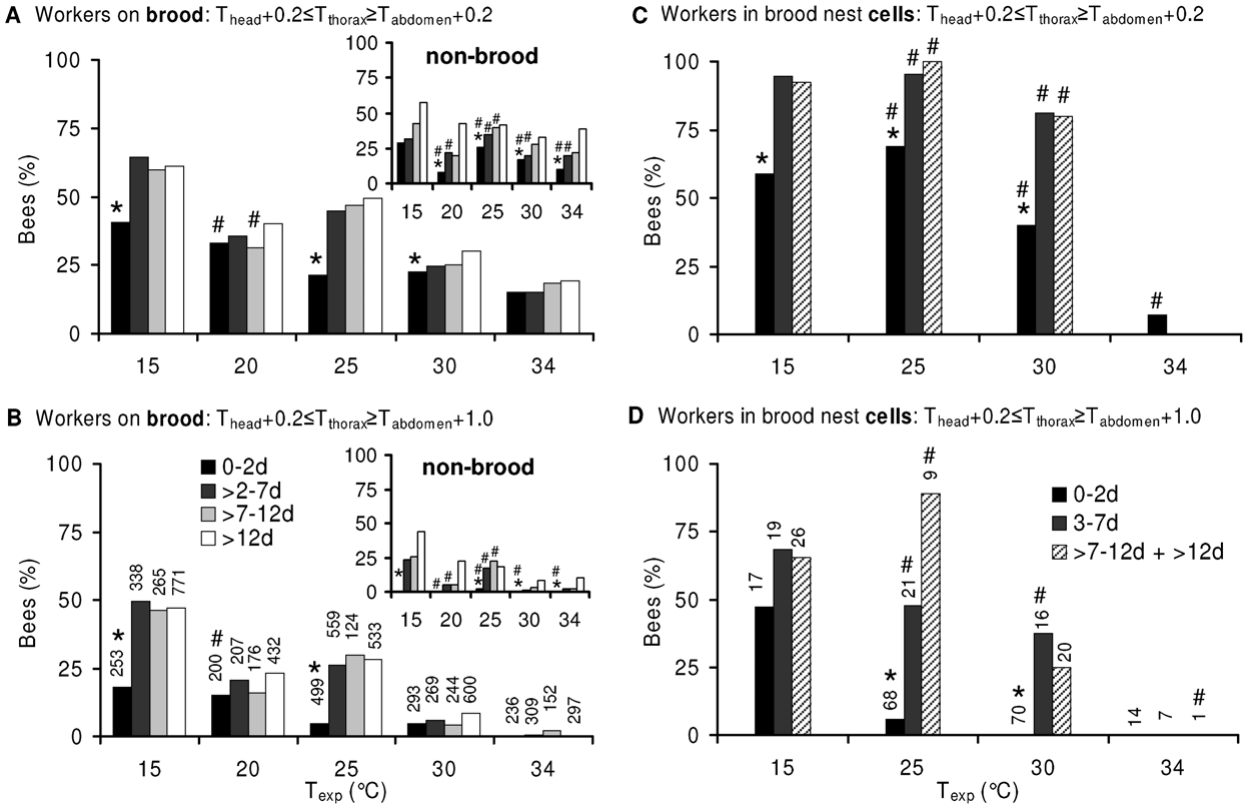
Percentage of endothermic bees in dependence on thermal stress (T_exp_). Shading of bars  =  age. (A-B) Percentage of actively heating workers on brood (main graphs) and non-brood areas (inserts); (C-D) percentage of actively heating workers in open brood nest cells. Parts (A, C) and (B, D) display different levels of endothermy (T_thorax_-T_abdomen_). Number of investigated workers of each age class ( = 100%) written above each column in (C) and (D). Number of measurements for non-brood areas (inserts) with increasing age: T_exp_ = 15°C: 14, 47, 140, 971; T_exp_ = 20°C: 12, 127, 157, 651; T_exp_ = 25°C: 85, 227, 125, 698; T_exp_ = 30°C: 72, 178, 172, 562; T_exp_ = 34°C: 68, 189, 163, 776. Statistics: *, differences significant (P<0.05, chi^2^_3.841_ >3.94) between 0–2 d old and >2 d old bees at a certain T_exp_ (at T_exp_ = 20°C, P<0.1, chi^2^_2.706_ = 3.484). #, differences *not* significant (P>0.05) between T_exp_ = 15°C and T_exp_ >15°C in each age class; otherwise P<0.05 (chi^2^_6.239_ >6.94 in A, B and chi^2^_5.731_ >7.39 in C, D).

In order to get an overview of the number of reliably endothermic bees on the combs we filtered those bees out where T_thorax_ was not only higher than T_abdomen_ but also at least 0.2°C higher than T_head_: T_head_+0.2°C ≤ T_thorax_ ≥ T_abdomen_+dT (dT = +0.2 or +1.0°C; Fig. 5). The percentage of probably endothermic bees on the combs increased with cold stress in all age classes and at all investigated levels of endothermy on the brood nest as well as outside it (Fig. 5A-B). On the brood nest, at T_exp_ = 15°C 18.2% (46 of 253) of the young bees (≤2 d) and 47.6% (654 of 1374) of the bees older than 2 days were endothermic at the dT >1.0°C level. On the other extreme of experimental temperatures (T_exp_ = 34°C), this percentage was reduced to 0.0% (0 of 0) in the young and to 0.5% (4 of 758 bees) in the older bees (statistics in Fig. 5). Young bees (0–2 d) were less often endothermic than the older ones at T_exp_ = 15 and 25°C, and showed a lower tendency at T_exp_ = 20°C. This tendency seemed to be more pronounced outside the brood nest (inserts in Fig. 5A-B) than on it.

Inside brood nest cells (Fig. 5C-D), at T_exp_ = 15°C, 66.7% (30 of 45) of the bees older than 2 days were endothermic at the dT >1.0°C level. At T_exp_ = 34°C, this percentage was 0.0% (0 of 8 bees). Young bees in cells (0–2 d) were endothermic at the dT >1°C level at a rate of 47.1% (8 of 17) and 0.0% (0 of 14) at T_exp_ = 15 and 34°C, respectively (statistics in Fig. 5).

A comparison of endothermic activity between bees inside cells and on combs on the basis of the above thermal classification has to consider that, if T_thorax_-T_abdomen_ is used as a correlate of endothermic activity, local thermal gradients might be higher for bees in cells and lead to an overestimation of their endothermic activity. To compare these local ambient gradients, reliably ectothermic bees which are unable of heating the thorax (0–24 h old) can be used as gradient thermometers. A correlate of the ambient gradient along the body is the T_head_-T_abdomen_. In the brood nest, even at very high thermal stress (T_exp_ = 15°C), it was not significantly different between bees in cells and on combs despite a visible trend (1.12±1.05°C in cells *vs*. 0.49±0.84°C on combs; n = 8 *vs.* 79; P = 0.142, t test). At moderate thermal stress (T_exp_ = 25°C) gradients of T_head_-T_abdomen_ were nearly identical (0.11±0.35°C in cells *vs*. 0.09±0.35°C on combs; n = 43 *vs.* 274; P = 0.732).

The rate of bees classified as endothermic at the dT >1°C level was not different between bees in brood nest cells (Fig. 5B) and on combs (Fig. 5D) at T_exp_ = 34°C (chi^2^_3.841_ = 0–0.02). Heating at this T_exp_ was extremely rare both inside and outside cells in all age classes (0–2 d, 3–7 d, ≥7 d tested). At lower T_exp_ the 3–7 and >7 days old bees were always more often classified as endothermic in the cells than on the combs (Fig. 5). Differences were significant at a T_exp_ of 30 and 25°C (P<0.05; chi^2^_3.841_ = 4.76–21.1). At a T_exp_ of 15°C the difference was weakly beyond significance in the ≥7 days old bees (P<0.1; chi^2^_3.841_ = 3.47), and not significant in the 3–7 days old bees (chi^2^_3.841_ = 2.52). The young bees (0–2 d) were more often endothermic in the cells than on the combs only at T_exp_ = 15°C (chi^2^_3.841_ = 9.16) but not at higher T_exp_.

### Local Thermal Gradients

**Figure 6 pone-0008967-g006:**
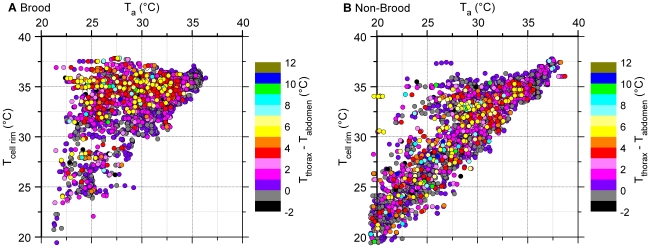
Intensity of endothermy (T_thorax_-T_abdomen_) of bees on combs in relation to individual bees' local ambient temperatures (T_cell rim_ and T_a_). See Fig. S1 for correlations between T_cell base_ and T_cell rim_, and between T_a_ and T_exp_.

**Figure 7 pone-0008967-g007:**
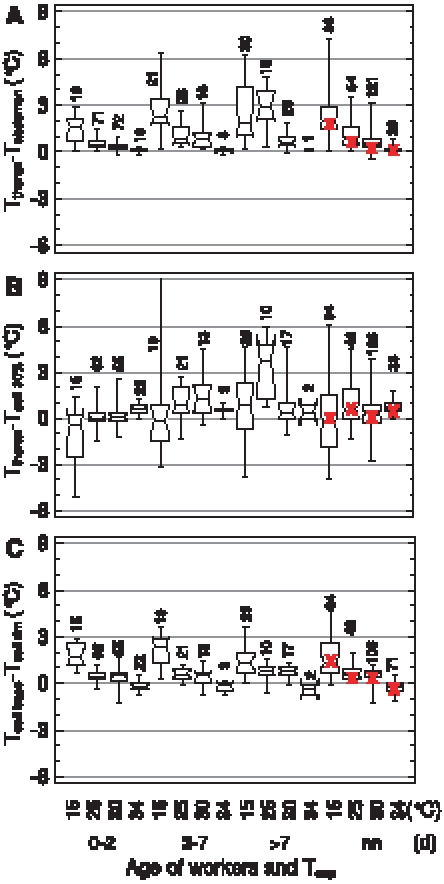
Thermal relations in open brood nest cells visited by bees. Box plots: median with 1^st^ and 3^rd^ quartile, maximum and minimum. Notches which do not overlap indicate significant differences (P<0.05). nn  =  unmarked workers; red crosses  =  medians of all age groups of marked bees (not significantly different from nn bees, except T_thorax_-T_abdomen_ at T_exp_ = 25 and 30°C, P<0.05, U test).T_cell 30%_ is the cell wall temperature at the estimated mean contact position of the thorax with the cell wall at 30% cell depth as measured from the base, calculated by linear interpolation between T_cell base_ and T_cell rim_. Numbers  =  measurements (bees).

We found highly significant correlations between T_a_ and T_exp_, and between T_cell base_ and T_cell rim_ (Table 1, Fig. S3). A comparison between brood and non-brood areas showed that these gradients were influenced by the bees' endothermic activity (Fig. S3). The effect of endothermy on thermal gradients was especially pronounced for the relation of T_cell rim_ and T_a_, which could not be described by a simple linear regression (Fig. 6). The temperature gradient in cells (median T_cell base_-T_cell rim_) increased with increasing cold stress. In empty cells not visited by bees it was −0.1, 0.4, 0.5, and 1.3°C at T_exp_ = 34, 30, 25 and 15°C, respectively. In cells visited by unmarked bees it was quite similar, amounting to −0.2, 0.7, 0.6 and 1.4°C (Fig. 7C), respectively. There was no statistical difference between not visited and visited cells (P≥0.079, Mann Whitney U test) except at T_exp_ = 30°C (P<0.001). The median cell temperature gradients were −0.25, 0.5, 0.4 and 1.7°C in cells visited by 0–2 days old bees, −0.1 0.5, 0.6 and 2.4°C in cells visited by >3–7 days old bees, and −0.4, 0.8, 0.8 and 1.3°C in cells visited by ≥7 days old bees, respectively (Fig. 7C).

**Table 1:** Regressions between hive temperatures and experimental temperature.

Regressions	R	SD (reg.)	n	P <
Brood				
T_cell base_ = 3.66875+0.90802*T_cell rim_	0.89924	1.008	5951	0.0001
T_a_ = 22.5267+0.33709*T_exp_	0.71768	2.059	7072	0.0001
Non-brood				
T_cell base_ = 0.05452+0.99284*T_cell rim_	0.97831	0.945	5249	0.0001
T_a_ = 14.38952+0.60827*T_exp_	0.86468	2.42054	5672	0.0001

For the relation of T_cell rim_ and T_a_ see Fig. 6. T_a_  =  local air temperature near the bees, T_exp_  =  experimental (environmental) temperature, SD (reg.)  =  standard deviation around regression lines, n  =  number of measurements.

In unmarked bees inside brood nest cells, which had emerged naturally in the test colonies (‘nn’ in Fig. 7A), the median gradient of T_thorax_-T_abdomen_ increased from just 0.1°C at T_exp_ = 34°C to 2.0°C at T_exp_ = 15°C. In parallel, the variation of T_thorax_-T_abdomen_ increased considerably. The range of central 50% (1^st^ - 3^rd^ quartile), for example, increased from 0.2 at T_exp_ = 34°C to 1.5°C at T_exp_ = 15°C, the total range from 1.0 at T_exp_ = 34°C to 7.2°C at T_exp_ = 15°C. Fig. 7A also shows that the increase with cold stress was smaller in young bees (0–2 d: 0.1 to 1.6°C) in comparison to the older ones (>2 d: 0.1 to 1.9–2.3°C; comparison of 0–2d old with >2d old bees: P<0.05 at T_exp_ = 15°C, P<<0.0001 at T_exp_ = 25 and 30°C, P = 0.67 at T_exp_ = 34°C; Mann Whitney U test).

### Heat Transfer between Bees in Cells and Combs

**Figure 8:**
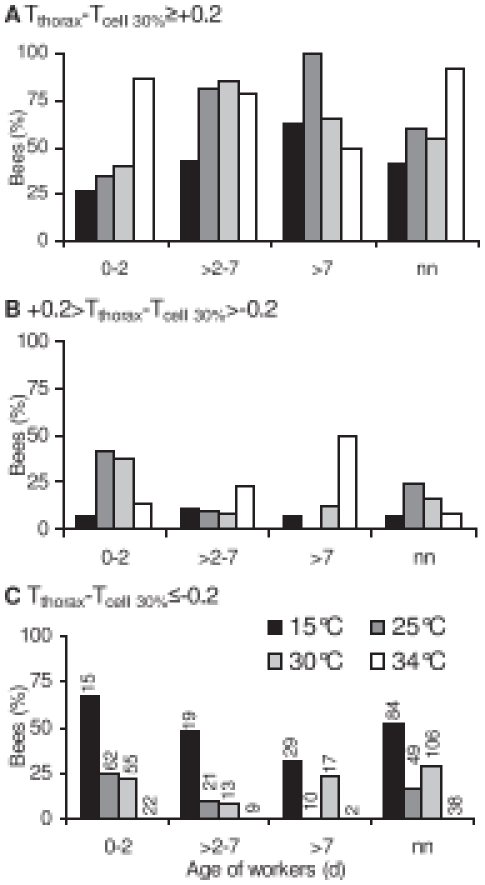
Heat transfer between bees in cells and brood combs. Percentage of workers of different ages in brood nest cells with (A) higher T_thorax_ than T_cell 30%_, (B) approximately equal T_thorax_ and T_cell 30%_, and (C) lower T_thorax_ than T_cell 30%_, in dependence on thermal stress (T_exp_  =  shading of bars). Sample numbers in (C) represent 100%. nn  =  naturally emerged, unmarked bees. nn bees did not differ from the averaged age classes of marked bees (P>0.1; Chi^2^_2.706_ = 0.00–2.143) with exception of bees at T_exp_ = 30°C in (B) (P<0.05; Chi^2^_3.841_ = 4.163).

A considerable amount of the difference of T_thorax_-T_abdomen_ was caused by the temperature gradient in the cells (compare Fig. 7A with Fig. 7C). The center of honeybee endothermy is the thorax. We estimated the position of contact between the thorax (the heat source during endothermy) and the cell wall to be at about 30% of the cell depth as measured from its base on average. In order to judge the direction of the heat flow between bees and combs, we calculated the difference between thorax and its cell contact position (T_cell 30%_) by interpolating the temperature between cell base and cell rim linearly (Fig. 7B). If the test colonies were not exposed to cold stress (T_exp_ = 34°C) the majority of the bees in brood nest cells was warmer than the cell wall (Fig. 8A), and there were no differences between the age groups (P>0.05; Chi^2^_3 comparisons_ = 0.35–1.75<5.731). At this T_exp_, however, the presence inside cells was generally low (Fig. 3B). The unmarked bees in the cells had a median T_thorax_ of 0.65°C above T_cell 30%_ at T_exp_ = 34°C but of 0.34°C below T_cell 30%_ at T_exp_ = 15°C (‘nn’ in Fig. 7B). Variation of central 50% increased from 0.5 to 3.4°C, and the total range from 1.8 to 6.0°C. The age analysis showed that predominantly the young bees (0–2 d) became cooler (−0.44°C) than T_cell 30%_ at the lowest T_exp_ of 15°C. In older bees (>2–7 d) less individuals had a T_thorax_ below T_cell 30%_. The majority of bees older than 7 days had a T_thorax_ higher than T_cell 30%_ at T_exp_ = 15°C (Fig. 7B).

The rate of bees inside cells being warmer than the cell wall (Fig. 8A) was not different between the >2–7 days old and the >7 days old bees (P>0.05; Chi^2^_3 comparisons_ = 0.64–2.19<5.731; all T_exp_). At T_exp_ = 25 and 30°C, 0–2 days old bees transferred less often heat to the comb than the combined older age classes (P<0.01; Chi^2^ = 10.14 and 8.63>6.635). At T_exp_ = 15°C the visible trend could not be proved statistically (P<0.1; Chi^2^ = 3.46<3.841). The unmarked bees in cells (‘nn’), which had emerged naturally in the observation hives, serve as an independent corroboration for the reliability and unbiased nature of these results. They showed the same dependence on T_exp_ as the marked bees both concerning body temperature relations (Fig. 7) and probability of heat transfer to the combs (Fig. 8).

## Discussion

### Mechanisms of Thermal Homeostasis

The honeybees' endothermic activity is one of the main factors to establish thermal homeostasis in a colony. In insects capable of endothermy, the flight muscles are the source of active heat production [e.g. 1], [44], [48], [49]. Since endothermy increases the thorax temperature in relation to the abdomen, the temperature difference between thorax and abdomen (T_thorax_-T_abdomen_) is a good correlate of endothermic activity. In our experiments T_thorax_-T_abdomen_ of individuals was strongly affected by T_exp_. As the thorax temperature excess T_thorax_-T_abdomen_ is always a mixture of local temperature gradient and degree of endothermic heat production, more clarity on endothermic activity can be achieved by filtering those bees out where the thorax was the warmest body part. Such analysis shows that the frequency of endothermic bees is higher on the brood nest than in non-brood areas, especially at extreme cold stress (Fig. 5, 6). However, there is always a considerable number of endothermic bees outside the brood nest. These bees contribute indirectly to thermal homeostasis of the brood because a minimized temperature gradient means a reduced heat flow out of the brood nest. Foragers, which are always endothermic during their returns to the colony [50]–[53], also contribute to this reduction of temperature gradients though they preferably stay in peripheral comb areas near the entrance during daytime [54], [55]. During night-time, by contrast, foragers were observed to cluster in peripheral hive areas. Though the majority of them is at rest [56] and not endothermic in a standard colony (our own unpublished observations), their high number nevertheless provides a considerable amount of resting heat production [31]. In addition, their increased number at nighttime contributes to colony isolation.

It has to be kept in mind that a T_exp_ of 15°C in our 2-comb observation hives represents a much higher thermal stress for the bees than in standard colonies. The heat loss of standard colonies is essentially smaller because of the parallel arrangement of several combs, better isolation, a higher number of bees, and (usually) less external convection than in our experiments. We estimated an environmental temperature (T_exp_) of about 0–10°C to initiate an extent of endothermy in standard colonies comparable to our observation hives at T_exp_ = 15°C (our own unpublished experiments; [49]).

#### Active compensation of heat loss

Our measurements showed that from low to high cold stress (T_exp_ = 30 to 15°C) a considerable number (mostly noticeably more than 50%) of the bees on the combs were cooler than the comb surface (T_cell rim_; Fig. 4D). In addition, the surface of open cells was cooler than their base (Fig. 7; see also [10]). This means that in many locations the heat flow is from the combs to the bee ways (intercomb spaces) and into the ectothermic bees there. To achieve thermal constancy of the brood this heat loss has to be compensated and minimized. Compensation is done by the endothermic bees on the comb surface and inside open cells (Figs 4–8; [9], [10]), and for the smaller part also by drones [57]. It might seem strange that average thorax temperatures of bees on combs (in intercomb spaces) are below the cell rim temperatures at low environmental temperatures, as the bees are the main source of the heat. However, thermal gradients may still persist as long as the heat production of the larvae and pupae themselves suffices to compensate for the heat loss. The duty of the bees is to minimize but not completely remove thermal gradients. Gradients have just to remain stable. If larvae are young and thus small, compensation by the bees has to be increased. Even more, heat production of the brood necessitates maintenance of (small) thermal gradients. If environmental temperature (T_exp_) is 34°C, where gradients in cells already reverse (Fig. 7), bees already spread water on combs to get rid of excess larval heat [24], [49].

The intensity of endothermy, however, varies in a wide range, and many bees are only weakly endothermic (Fig. 4). Our observations on endothermic reactions represent a mean of general trends. Actually, individual bees do not heat continuously but show heating bouts lasting several minutes, alternating with ectothermic phases [10]. The differential decisions of individual bees to heat or not is suggested to be determined by temperature threshold differences between individuals, according to the threshold model of task allocation in polyandrous social insect colonies [e.g. 58]. A higher genetic diversity (with a higher probability of differing thresholds) was reported to improve thermal homeostasis of honeybee colonies [27]. We suggest that individual endothermy is kept up until an individual optimum of local temperature (probably T_comb_, see below) is achieved. The sum of temporally and spatially distributed individual phases of endothermy enables the superorganism honeybee colony to achieve thermal homeostasis.

Active (endothermic) compensation of heat loss is done by relatively few bees. On the basis of an estimation of the relation between thorax temperature excess above ambient air (T_thorax_-T_a_) and oxygen consumption in endothermic bees [59], [60], and measurements of the resting metabolism [30], [31] a rough estimation is possible how much heat an endothermic bee dissipates in comparison to ectothermic bees. An endothermic bee with a temperature excess (T_thorax_-T_a_) of 1°C above the resting level (at T_a_ ∼34°C) produces roughly 2–4 times the heat of resting older bees (>2 d), and approximately 4–6 times the heat of freshly hatched bees (<12 h). A temperature excess of 5°C compensates for the resting heat production of approximately 11–16 bees capable of endothermy (>2 d), and of 16–23 young bees (<12 h) which are not able of endothermic heat production, respectively. So heat production of a few endothermic bees eases much burden of many nestmates. Their heat surely has a considerable effect on local ambient temperature [compare 9]. This affects local comb temperature most if bees heat cells from within [10].

The thermal activity of endothermic bees does not only apply heat directly to the surrounding air and the combs but has also an important reinforcement effect on passive heat production of the ectothermic bees: it shifts them to a higher basal metabolic rate along their curve of resting metabolism [29]–[31], [61]. The same holds for the larvae [21]. In Fig. 6 this is represented by the fact that the distribution of endothermic bees bases upon a large ‘carpet’ of bees with no or just weak active heat production.

#### Passive minimisation of heat loss

Heat flow minimisation is achieved by the presence of bees in the bee ways and inside of part of the open cells. They not only reduce the convective but also the diffusive heat loss by reduction of the thermal gradient. The resting metabolism is, because of the high number of ectothermic bees, an important parameter for heat flow minimisation and compensation.

#### Heat transfer in cells

Usually, many of the open brood cells are not visited by bees for longer periods of time. A high presence inside cells was only observed after increased emergence the day before. Most of these bees are young (Fig. 3B) and therefore not capable of much endothermy [62]. Of the older bees it is mainly the nurses (3–12 d old) which visit open brood cells, for controlling and feeding the larvae, and for active heat production (Figs 5, 7, 8).

The temperature gradient in brood nest cells (T_cell base_-T_cell rim_) increased significantly with increasing cold stress (decreasing T_exp_; Fig. 7C), but we did not find a statistical difference between empty and visited cells. This, however, would not justify the conclusion that visitation has no effect on cell temperature. Rather, visitation may have compensated for temperature differences. Direct thermographic observations demonstrate this [10]. At T_exp_ = 34°C, the average (median) heat flow was always directed inwards, from the bee ways to the comb (T_cell base_–T_cell rim_ = −0.25°C; Fig. 7C). At lower T_exp_ the average heat flow was always outwards. We suggest the equilibrium environmental temperature (T_exp_) of our observation hives concerning heat flow in visited open brood cells to have been at about 32.5–33°C (see Fig. 7C). In standard colonies we suggest it to be below this level because of a better volume to surface ratio, better insulation and less internal convection because of the parallel arrangement of the combs.

If it is to judge the heat flow inside visited cells in more detail, the difference between the thorax (the source of heat production) and the cell wall is of particular interest. Inside cells rather weak endothermy may reverse the direction of heat flow. At high cold stress the median T_thorax_ especially of the 0–2 days old bees was below the cell temperature at the point of contact with the cell wall (T_cell 30%_, Fig. 7C). In these cases the heat flow was from the cell wall to the thorax (Fig. 8). As the older bees (>2 d) were more frequently endothermic, the direction of heat flow was in more bees directed from the thorax to the cell wall. It has to be kept in mind, however, that these considerations refer to a value of T_cell 30%_ interpolated between T_cell base_ and T_cell rim_. This works best in ectothermic bees. In the case of endothermic bees in cells, the gradient between cell base and cell rim is probably not linear. Somewhat higher temperatures than calculated by interpolation have to be expected at the contact site of a heated thorax with the cell wall [10]. Nevertheless, as the heat applied to the contact site distributes within the cells in time, our estimation describes the average heat flow inside visited cells. Bees inside cells, however, contribute to comb temperature stabilisation not only in the endothermic but also in the ectothermic state. Ectothermic bees are not just passive insulators which reduce convective heat loss due to their presence. In the narrowness of a cell, their rather high resting metabolism (and thus heat production) [31] at brood comb temperature additionally reduces the thermal gradient and thus the heat flow out of the cells. Heat transport from the cells to the bee ways via respiration is suggested to be of minor importance (though not negligible) in ectothermic bees, because at T_a_ = 30 and 34°C (as is usual on a brood nest), resting bees breath only every 37 and 28 seconds on average, respectively [31], and because the temperature gradient inside cells is rather small anyway at low to moderate temperature stress (Fig. 7A).

### Age Polyethism

Behavioral analysis of temporal polyethism within honeybee colonies usually addresses task allocation in terms of the visually observable activity like cell cleaning (days 1–3), nursing (days 4–12), food processing and nest maintenance (days 13–20), and foraging (days >20) [38]–[43]. Harrison [32] was the first to investigate the temporal polyethism of workers concerning active heat production of individual bees for the maintenance of thermal homeostasis. He reported that, under very high cold stress (one single comb exposed to 15°C), he was not able to find age-dependent differences in the degree of endothermy in bees older than two days. Only 1-day-old bees showed a smaller increase of T_thorax_ above T_a_. In our experiments this was best visible with the temperature excess of T_thorax_-T_abdomen_ (Fig. 4B). Especially the youngest bees (<24 h) showed no or just a very weak endothermic activity both in terms of intensity and percentage of endothermic workers on the combs (Figs 4, 5). We confirm the finding of Harrison [32] that the nurses do not differ from middle aged bees and foragers. This finding, however, might have resulted from the extreme cold stress in those experiments, forcing more age divisions to take part in active heat production as usual. Our graduated manipulations of cold stress did not result in different age distributions of endothermic activity. The same holds for drones, though their presence on the brood nest is considerably lower in comparison to the workers [57]. In addition, our experiments show for the first time that this not only applies for workers on the combs but also for bees in cells. The rate of bees inside cells being warmer than the estimated cell wall temperature (T_cell 30%_), i.e. the number of bees transferring heat to the combs, also was not different between the age groups older than two days. Only the youngest bees (0–2 d) showed a lower probability of heat transfer to the combs (Fig. 8A).

Concerning active heat production, therefore, the four classical divisions of task allocation reduce to only two: 0 to ∼2days and older. The shift between these two task divisions is gradual as is common in honeybee polyethism, starting at an age of about 24 hours and being finished at about 5 days (Fig. 4). This resembles the age dependent development of endothermic ability [62]. Task allocation concerning endothermy, therefore, directly depends on physiological ability. This has the consequence that the passive effect of cold stress is highest in very young bees, whereas the bees' physiological (endothermic) reaction to cold stress is highest in older bees. Young bees compensate for their inability of active heat production by behavioral mechanisms, i.e.by their increased preference of the warm brood nest ambience (Fig. 3A, [63]) and their tendency to enter empty cells (Fig. 3B). As endothermic capacity increases in the nurse age (3–13 d, [62]) the brood nest preference decreases (Fig. 3A, Fig. S1). Bees engaged in hive maintenance and food storing (>13–22 d) are already as abundant outside the brood nest as on it. All ages of honeybees can be seen as ‘active isolators’ with respect to environmental changes. The young ones preferably in terms of migration activity into or out of brood nest cells (Fig. 3B, insert), and the older bees both by migration activity to or from the brood nest (Fig. S1; [49]) and additionally in terms of active reduction of thermal gradients by endothermy.

In conclusion it can be said that thermal homeostasis in honeybee nests is the result of the cooperation of *all* colony members and all temporal castes of adults. This includes heat production by the larvae and pupae [19], [21], passive worker and drone heat production via the resting metabolism [30], [31], [64], active (endothermic) heat production by hive bees and drones on the combs [9], [57, this paper] and inside cells [10, this paper], active heat production by foragers [48], [50]–[53], migratory movements of workers on the combs (changes of bee density; this paper and [49]), cooling of combs by foragers [22], [24] and via social control of colony ventilation [23], and heat shielding [65], [66].

### Individual Development of Endothermy

Our measurements showed that especially the very young bees of 0–2 days age seek the warm area of the brood nest (Fig. 3A). This preference is less pronounced in 3–7 days old bees, and disappears completely in older workers. If empty cells are available it is predominantly the very youngest bees which enter them (Fig. 3B). These (young) bees are usually thought to enter the cells to clean them [e.g. 37], [38]. However, in search for the causes that govern the young bees' preference for the brood nest and the empty cells, one has to look beyond arguments of social behavior, and additionally consider physiological necessities. From a physiological point of view bees are not adults after emergence. They are not yet capable of proper activation of their flight muscles both for flight and endothermic heat production. Capability of endothermy develops only within the first days after emergence [44], [62]. Morphological and enzymatic make-ups are completely developed only at an age of about 8–9 days [67]–[71]. As freshly emerged bees are poikilothermic [44], their respiratory turnover, which is the basis for development, is strongly temperature dependent [30]. We suggest the young bees to seek the high brood nest and cell temperature for self-warming to guarantee proper development.

### Stimuli of Endothermy

We suggest the individual decision of bees to heat or not to depend on their local environment, i.e. T_comb_ and/or T_a_, but most probably not on the outside temperature (T_exp_). We do not know, however, whether the ‘local environment’ is the actual environment, or whether bees make a temporal integration on their way across the combs. Recent work has shown that especially the older age classes (‘middle aged bees’, 13–20 d) show considerable migration activity, presumably also for information sampling, beside search for work [43].

Only suggestions exist about what bees measure, comb or air temperature. Besides measuring comb temperature they might just regulate T_a_ at a level which guarantees a minimum heat flow away from the combs. From experiments with combs cooled with a heat exchange plate, Kronenberg and Heller [20] suggested the comb temperature to be the stimulus. Bujok et al. [9] reported intense antennal contact with the comb surface of endothermic workers pressing their body on the comb surface to facilitate heat transfer. An analysis searching for the stimulus temperatures for the release of active heating which can be perceived by bees, has to take into consideration that there exists a chain of interdependence between T_cell base_, T_cell rim_, T_a_, and T_exp_. While we found direct correlations between T_cell base_ and T_cell rim_ and between T_a_ and T_exp_ (Table 1, Fig. S3), the interrelation of T_cell rim_ and T_a_ at the bees' positions was more complex, especially on the brood nest (Fig. 6). This ‘complexity’ is caused by the bees' endothermic counteraction to stabilize brood temperature as a reaction to changes of environmental temperature (our manipulations of cold stress). On the brood nest, there was a clear concentration of strongly endothermic bees (T_thorax_–T_abdomen_ >3°C) around a T_cell rim_ of about 35°C, whereas along the T_a_ axis endothermy was more evenly distributed. We conclude that the attempts to stabilize T_cell rim_ directly were higher than to stabilize T_a_, which is supported by observations of direct heat transfer to the comb surface [9] and to the cell interior [10]. Our data show that bees inside brood nest cells are more often endothermic than bees on the surface of brood combs (Fig 5). This supports the hypothesis that bees undertake special efforts to stabilize comb temperature by applying heat as near to the brood as possible. Therefore, we suggest the bees to react to comb temperature first of all and to air temperature in the second place.

Comparison of endothermic activity between brood and non-brood areas (Fig. 6) and literature reports on the relation of brood and non-brood temperatures [13], [18], [20] suggest that accurate regulation of brood temperature requires a trigger of higher order to coordinate endothermic activity and behavioral reactions between colony members. In addition to temperature, therefore, brood pheromones are suggested to play an important role in brood temperature homeostasis [13].

### Conclusion

Thermal homeostasis of honeybee colonies is achieved by a combination of active regulatory processes and passive effects. The central parameter of active processes is “endothermy on demand” of adult bees (older than about 2 days), which has an important reinforcement effect on passive heat production of the many ectothermic adult (>∼2 days) and young (<∼2 days) bees, as well as of larvae and pupae. The second foundation of thermal homeostasis is behavior. This includes migration activity within the colony (changes of bee density), evaporative cooling with water, and regulation of internal heat transport via convection (fanning).

## Supporting Information

Figure S1 Distribution of workers of different ages (A-J) between brood (open and sealed) and non-brood areas in dependence on age and thermal stress (Texp). Mixed brood additionally classified in young bees (2–54 h old) (A - E). Numbers  =  n = 100% of columns.
(7.51 MB EPS)

Figure S2 Body temperature of workers of different ages on non-brood areas in dependence on thermal stress (Texp). Box plots: median with 1st and 3rd quartile, maximum and minimum of (A) Tthorax, (B) Tthorax-Tabdomen, (C) Tthorax-Ta, and (D) Tthorax-Tcell rim. n  =  measurements (bees), low sample sizes of 1 or 2 are indicated.
(1.82 MB EPS)

Figure S3 Correlations of temperatures in observation hives. (A, B) Correlation of cell temperatures. (B, C) Correlation of individual bees' local ambient temperature (Ta) and environmental temperature (Texp). See Fig. 6 for relation of Tcell rim and Ta.
(1.06 MB TIF)
