# Supplementary figures and images for: Honeybee Colony Thermoregulation – Regulatory Mechanisms and Contribution of Individuals in Dependence on Age, Location and Thermal Stress
Source: PLoS One. 2010 Jan 29;5(1):e8967. doi: 10.1371/journal.pone.0008967 (PMC2813292; doi:10.1371/journal.pone.0008967)

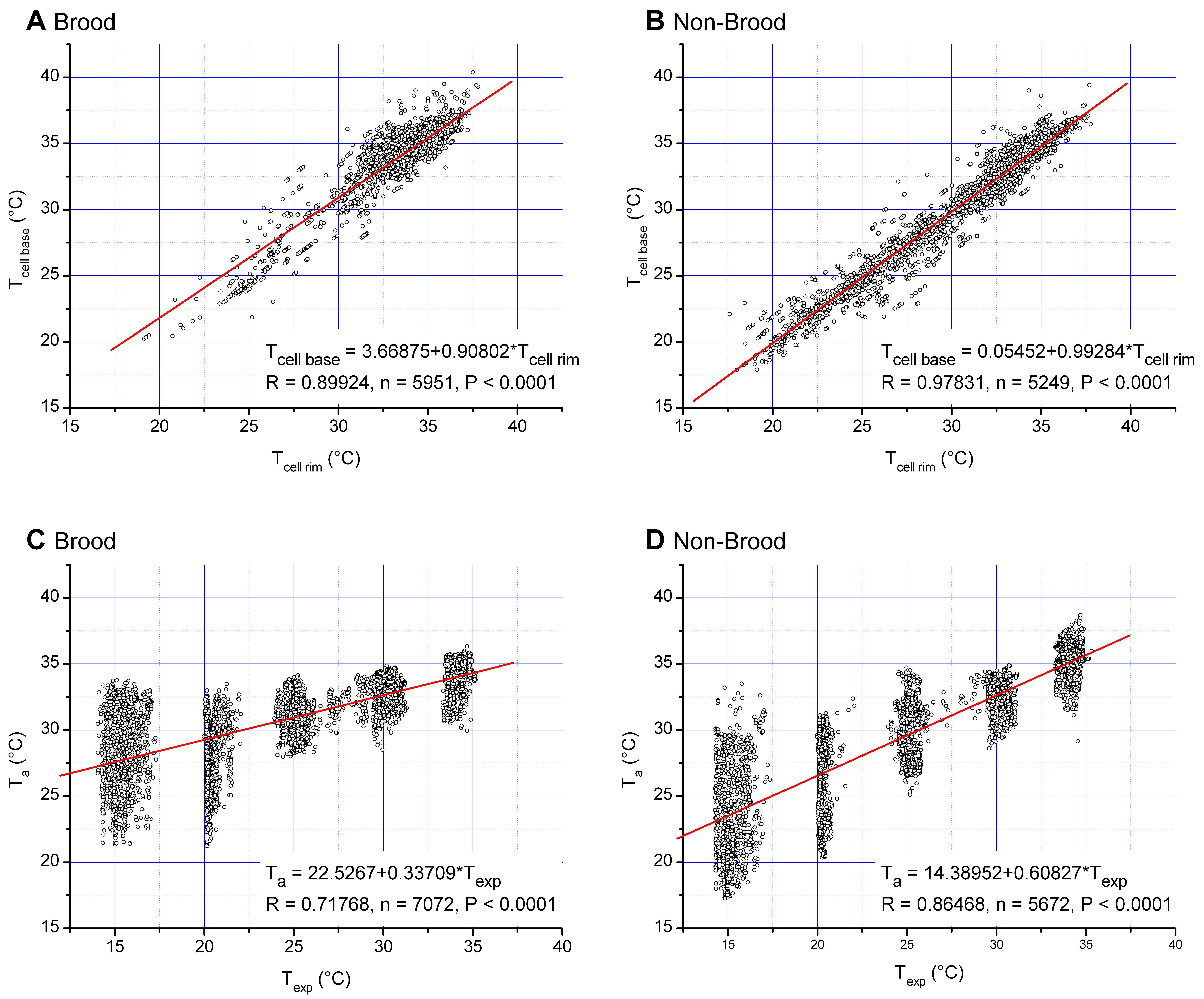

Supplement: Figure S3 — Correlations of temperatures in observation hives. (A, B) Correlation of cell temperatures. (B, C) Correlation of individual bees' local ambient temperature (Ta) and environmental temperature (Texp). See Fig. 6 for relation of Tcell rim and Ta. (1.06 MB TIF) [file pone.0008967.s003.tif]
